# A Fatal Case of Lisinopril-Induced Acute Necrotizing Pancreatitis

**DOI:** 10.7759/cureus.40071

**Published:** 2023-06-07

**Authors:** Omar M Masarweh, Feras Al-Moussally, Juan Pablo Meruvia Garron, Anuj Kunadia, Olga Karasik, Abdo Asmar

**Affiliations:** 1 Internal Medicine, University of Central Florida College of Medicine, Orlando, USA

**Keywords:** lisinopril-induced pancreatitis, fatal outcome, lisinopril, acute necrotizing pancreatitis, severe pancreatitis

## Abstract

Angiotensin-converting enzyme inhibitors (ACE-I), such as lisinopril, are used as first-line therapy in the treatment of hypertension, heart failure with reduced ejection fraction, and proteinuric chronic kidney disease due to their beneficial effects on reducing morbidity and mortality. Commonly cited adverse effects of lisinopril include hyperkalemia, acute kidney injury, and angioedema, and while uncommon, there have been reports of lisinopril-induced necrotizing pancreatitis in the literature. The true incidence of drug-induced pancreatitis is unknown since establishing a causal relationship between medication’s adverse effects and disease occurrence is difficult; however, there are validated tools such as the Adverse Drug Reaction Probability Scale that can aid in determining causality. Here, we present a case of a 63-year-old man with a history of hypertension who was being treated with lisinopril for eight months and developed a fatal case of lisinopril-induced severe necrotizing pancreatitis.

## Introduction

Acute pancreatitis (AP) is an inflammatory response of the pancreas that occurs due to a variety of reasons. The incidence and prevalence of AP in the United States have risen year after year with nearly 400,000 emergency room visits in 2014, an 18% increase from 2006 [1]. Often times the cause may not be identified, and this may contribute to AP being the fourth most common cause for hospital re-admission due to gastrointestinal, liver, or pancreatic conditions, accounting for over 390,000 hospital re-admissions in 2015 [1]. With a course that is difficult to predict, AP is a complex condition, with the majority of patients developing mild to moderate-severe disease and up to one-fifth of patients developing severe disease, which carries a mortality rate of roughly 20% [1].

The diagnosis of AP is usually made when two out of three criteria are met, including (A) abdominal pain consistent with AP, (B) elevated lipase above three times the upper limit of normal, and (C) imaging findings consistent with AP. Most commonly, AP can be attributed to gallstone obstruction, alcohol use, hypertriglyceridemia or may be idiopathic. Medication-induced AP is considered to be responsible for less than 5% of cases and is likely underreported [2]. Although it is difficult to establish a causal relationship, medications that have been implicated in causing AP include azathioprine, 6-mercaptopurine, valproic acid, and, rarely, angiotensin-converting enzyme inhibitors (ACE-I) [2]. Although data is limited to observational studies, there has been a reported 2.1-fold increased risk of developing AP with ACE-I use [3]. Additionally, there is no clear data regarding a dose relationship or differences between ACE-I types, thus indicating a class effect. Despite the vast uncertainty surrounding the pathophysiology and true incidence, this adverse effect is mentioned in the package insert [3]. Here, we present a case of a previously healthy man who presented with severe necrotizing pancreatitis that proved to be fatal and was determined to be likely caused by ACE-I lisinopril.

## Case presentation

A 63-year-old man with a history of hypertension treated with lisinopril for eight months presented to the emergency room with one day of a 6/10 non-radiating, achy epigastric abdominal pain with associated diffuse swelling, nausea, and vomiting. He had no reported history of alcohol or tobacco use, gallstones, trauma, new medication, weight loss, changes in stool, or skin discoloration. Initial vital signs showed sinus tachycardia of 132 beats per minute, blood pressure of 110/78 mmHg, and he was saturating 97% on room air. On physical exam, he was in mild distress due to abdominal pain with moderate tenderness to palpation in the epigastrium; however, without evidence of guarding or rebound, lung sounds were clear bilaterally and he was not in respiratory distress, and skin was warm and dry. Labs demonstrated leukocytosis, hemoconcentration with an elevated hematocrit, acute kidney injury with a high anion gap metabolic acidosis from pre-renal injury as well as lactic acidosis, hyperglycemia, elevated liver enzymes, lipase and lactate dehydrogenase, normal triglycerides, bilirubin, and calcium with an undetectable serum ethanol level (Table 1). Right upper quadrant ultrasound was negative for gallstones or gallbladder wall thickening. Computed tomography (CT) scan of the abdomen and pelvis without contrast revealed peripancreatic fat stranding and a moderate amount of free fluid around the pancreas with a prominent pancreatic head with ill-defined hypoattenuation. He received a two-liter bolus of normal saline and broad-spectrum antibiotics, and blood and urine cultures were obtained.

**Table 1:** Initial laboratory values on admission and hospital day three. *Abnormal.

	Admission	Day three	Reference range
White blood cell count (cells/mm^3^)	21,500*	15,900*	4-10.5
Hematocrit	61.8%*	46%	40.1-51
Sodium (mmol/L)	135*	134*	136-145
Potassium (mmol/L)	3.7	4.8	3.7-5.1
Carbon dioxide (mmol/L)	17*	20*	21-32
Serum creatinine (mEq/L)	3.55*	5.01*	0.55-1.3
Blood urea nitrogen (mg/dL)	30*	49*	7-18
Anion gap	22*	16.8*	10-14
Glucose (mg/dL)	341*	219*	74-106
Beta-hydroxybutyrate (mg/dL)	1.4		0.2-2.8
Calcium (mg/dL)	8.4	6.6*	8.8-10.5
Aspartate aminotransferase (u/L)	74*	111*	10-37
Alanine aminotransferase (u/L)	136*	64	12-78
Total bilirubin (mg/dL)	1.1	0.9	0.2-1.5
Lactic acid (mmol/L)	9.2*	3.6*	0.4-2.0
Lipase (u/L)	>15,000*		73-393
Triglycerides (mg/dL)	217*		<150
Lactate dehydrogenase (u/L)	699*		100-190
Serum ethanol	Undetectable		

**Figure 1:**
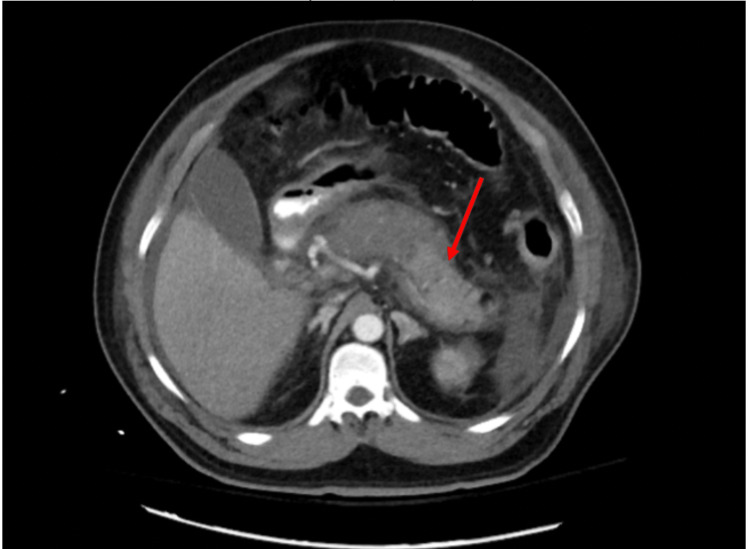
Computed tomography scan of the abdomen with contrast on day three showing edematous pancreas with areas of necrosis around the head, body, and tail (red arrow).

The following day, the patient developed acute hypoxic respiratory failure, presumably from acute respiratory distress syndrome, and was then switched to bilevel-positive airway pressure ventilation. Repeat labs showed a serum creatinine of 5.01 mg/dL, blood urea nitrogen of 49 mg/dL, white blood count of 15.9 cells/mm^3^, hematocrit of 46%, albumin-adjusted calcium of 6.6 mg/dL, and lactic acid of 3.6 mmol/L. Blood and urine cultures remained negative. After 48 hours of hospitalization, Ranson’s criteria score was calculated to be 9, which predicted a 100% mortality rate. On hospital day three, he went into multiorgan failure, requiring renal replacement therapy, vasopressors, and mechanical ventilatory support. A repeat CT scan of the abdomen with contrast was done looking for a possible abscess or area of walled-off necrosis that may be amenable to surgical removal; however, it showed pancreatic necrosis of the uncinate process, head, and proximal 1/3 of the body without a defined abscess (Figure 1). General surgery and hepatobiliary surgery teams were consulted and determined there was no opportunity for surgical intervention. To aid in the determination of the cause of his AP, an extensive workup including anti-neutrophil antibody (ANA), cytoplasmic antineutrophilic cytoplasmic antibody (c-ANCA), perinuclear antineutrophilic cytoplasmic antibody (p-ANCA), anti-mitochondrial antibody, complement components 3 and 4 (C3, C4), complement total (CH50), immunoglobulin G4 (IgG4), Mycoplasma IgM, HIV, Cytomegalovirus IgG and IgM, Coxsackie, COVID-19, hepatitis A, B, and C, ceruloplasmin, alpha-1 antitrypsin, IgG4, and ferritin were done and were unremarkable.

Unfortunately, his condition continued to deteriorate despite maximal ventilatory and pressor support. Goals of care discussion with the family was re-visited and care was transitioned to comfort measures and all aggressive medical management was discontinued. He quickly passed away after the removal of mechanical ventilation and pressor support.

## Discussion

Acute pancreatitis is an inflammatory disease involving the pancreas that is often mild and self-resolving with supportive care in about 80% of patients; however, complications may arise in up to 20% of patients leading to increased mortality [4]. Based on the Revised Atlanta Criteria for acute pancreatitis, severity can range from mild, moderate, or severe depending on organ dysfunction and systemic or local complications such as necrosis or abscess formation [2]. The most common causes of AP in developed countries are gallstones causing common bile duct and pancreatic duct obstruction and alcohol use, which combined account for about 70% of cases [2]. Other causes of AP are less common but include idiopathic (15-25%), hypertriglyceridemia (2-5%), drugs or medications (<5%), infections (<1%), and autoimmune (<1%) [2]. It is possible that many cases of AP deemed to be idiopathic are in fact medication-induced AP that was not diagnosed, which demonstrates the importance of being mindful of potentially implicated medications.

The pathogenesis of AP differs based on etiology and multiple different theories have been proposed over the years including pancreatic autodigestion, gallstone migration, enzyme activation, kinin and complement activation, necrosis, and pancreatic acinar cell apoptosis theories [5]. Each theory can explain the pathogenesis of specific cases, but no one theory helps capture all possible causes of AP, and in fact, most theories are still controversial. For example, for the most common cause of AP, gallstone obstruction, it is believed that a stone causes bile-pancreatic duct obstruction, increasing pancreatic duct pressure, bile reflux, and trypsin activation resulting in pancreatic auto-digestion, known as the common pathway or gallstone migration theory [6,7].

Medications such as azathioprine, 6-mercaptopurine, valproic acid, and ACE-I have all been implicated as potential causes of AP. In the case of ACE-I, proposed mechanisms include local angioedema, as ACE-I is known to decrease the degradation of bradykinin. The local angioedema obstructs the pancreatic duct leading to the entrapment of enzymes and possible pancreatic auto-ingestion [8]. This is supported by cases of hereditary angioedema-induced AP [9]. Additionally, it has been proposed that angiotensin II receptors contribute to pancreatic secretion and microcirculation, which may increase vascular permeability leading to angioedema and ductal obstruction. Furthermore, ACE-I has been known to cause hypoglycemia, which may contribute to causing AP via direct toxic effects on the pancreatic ducts [10].

It appears there is not a clear temporal relationship between ACE-I initiation and the development of AP, such as the case with angioedema. Furthermore, Kuoppala et al. investigated ACE-I and the risk of AP and found an increased risk of AP with ACE-I use but demonstrated no difference in the incidence between different ACE-Is indicating a potential class effect [3]. Other theories include direct toxic effects as well as upregulation of matrix metalloproteinase 9 (MMP-9). Chen et al. studied captopril in animal studies and showed that MMP-9 increases vascular permeability and increases the expression of transforming growth factor-beta, which may result in increased trypsin activation [11]. Interestingly, this association is not seen with angiotensin II receptor blockers (ARBs). A 2017 population-based study conducted by Bexelius et al. actually found a decreased risk of development of all forms of AP (mild, moderate, and severe) with ARBs (odds ratio of 0.77 (95% CI 0.69-0.86)) [12]. Their analysis also suggested a protective effect of ARBs against AP; therefore, it may be reasonable to prescribe ARBs for patients who developed AP due to ACE-I if they have a strong indication for renin-angiotensin-aldosterone system (RAAS) blockade such as cardiomyopathy or proteinuric kidney disease [12]. The exact etiology of this potential protective effect of ARBs is unclear; however, it is hypothesized that certain ARBs, such as losartan, may reduce inflammation in AP and reduce pancreatic hyperenzymemia [12].

Of the two reported cases in the literature of lisinopril-induced necrotizing pancreatitis, the medication was initiated weeks to months prior to being diagnosed with AP [13,14]. In our case, the patient was on lisinopril for eight months without complications or recent increases in dosage. In a 2020 systematic review of reported cases ranging from 1990 to 2019, there were 45 medications implicated in causing AP, including lisinopril and captopril [11]. These 45 medications were categorized as class 1a, meaning that alcohol, gallstones, hypertriglyceridemia, and other medications were ruled out as well as evidence of a positive re-challenge. Of these 45 reported medications, there were six cases of lisinopril-induced AP and three cases of captopril-induced AP [15]. Unfortunately, the timing of drug initiation with respect to the presentation of AP and severity was not reported for all cases. Additionally, the U.S. Food and Drug Adverse Event Reporting System (FDAERS) reported that between 1988 and 2022, there have been 406 cases of lisinopril-induced AP, of which 22 were fatal; however, specifics for each case are not available [16].

Certain tools have been created in order to aid in determining a causal relationship between an identified adverse event and a drug, one of which is the Adverse Drug Reaction Probability Scale (Naranjo scale) [17]. The Naranjo scale is a 10-question scale that is graded between (−4) and (+13) with scores of nine and above indicating a definite reaction, a score of five to eight indicating a probable reaction, a score of one to four indicating possible, and zero or less indicating doubtful. In our case, our patient’s characteristics gave him a score of 5, thereby indicating a probable interaction, meaning the reaction could not be reasonably explained by the known characteristics of the patient’s clinical state. Notably, this scoring system was designed to assess drug interactions causing liver injury, and certain aspects of this scoring system are not useful, such as detecting drug concentrations in the blood known to be toxic and whether the adverse event re-appeared after drug re-administration, both of which would not apply to our case. We believe that lisinopril was the cause of this patient’s AP since we effectively ruled out all other potential causes such as gallstones, alcohol use, hypertriglyceridemia, hypercalcemia, auto-immune, vasculitis, infectious and IgG4-related illnesses.

Our case is particularly unique in that it appears to be the first well-documented case of lisinopril-induced severe necrotizing AP that proved to be fatal. From our extensive PubMed review, only two cases of necrotizing pancreatitis attributed to lisinopril were reported, and in both cases, the patients did well. Our patient initially came in hemodynamically stable; however, quickly deteriorated transitioning from moderate edematous pancreatitis to severe necrotizing pancreatitis complicated by persistent multiorgan failure with acute respiratory distress syndrome requiring mechanical ventilation, acute renal failure requiring renal replacement therapy, and shock requiring high-dose vasopressors.

## Conclusions

This case highlights the importance of a few things. First, drug-induced pancreatitis can vary in severity from mild to severe and life-threatening. Second, the timing of drug initiation and the onset of adverse events is difficult to determine. In this patient, lisinopril was started eight months prior and without dose adjustments. Other cases of drug-induced pancreatitis echo a similar template in that establishing a temporal relationship is often difficult; however, it is most commonly seen with initiation and with up-titration of the dose. Third, our case highlights the importance of keeping a broad differential when determining the cause of AP, keeping in mind that the list of implicated medications is vast. Lastly, an accurate and comprehensive medication reconciliation at hospital admission is imperative; making sure to ask about all medications, when they were started, dose titrations as well as any over-the-counter medications or supplements. Reports like this are needed in order to conduct a larger meta-analysis of similar cases and better understand the pathophysiology and potential treatment options. Additionally, more research is needed to determine whether a dose-dependent association exists and potential risk-enhancing factors such as concomitant use of other AP-associated medications.
